# Treatment of AO Type C Fractures of the Distal Part of the Humerus through the Bryan-Morrey Triceps-Sparing Approach

**DOI:** 10.1155/2013/525326

**Published:** 2013-03-20

**Authors:** J. A. Fernández-Valencia, E. Muñoz-Mahamud, J. R. Ballesteros, S. Prat

**Affiliations:** Department of Orthopaedic and Trauma Surgery, Hospital Clínic, University of Barcelona, C/Villarroel 170, 08036 Barcelona, Spain

## Abstract

Several alternative approaches have been described to avoid the complications related to the olecranon osteotomy used to treat distal articular humerus fractures. The published experience with the triceps-sparing approach is scant. In this prospective study, a total of 12 patients with an articular humeral fracture were treated using this approach. At a mean followup of 1,7 years, the average range of motion was 112.8° (range from 85° to 135°); the elbow flexion averaged 125.5° (range from 112° to 135°) and the deficit of elbow extension 14.6° (range from 0° to 30°). All the elbows were stable. The Mayo Elbow Performance Score (MEPS) averaged 93.3 (range from 80 to 100). In the present series no failure of the triceps reattachment to the olecranon was found, and all the patients recalled returning to their previous daily life activities without impairment with a satisfactory MEPS. As a conclusion, the triceps-sparing approach can be considered for treating distal articular humerus fractures. We consider that three clinical settings can be more favorable to use this approach: those cases in which a total elbow prosthesis might be needed, cases of ipsilateral diaphyseal fracture, or presence of previous hardware in the olecranon.

## 1. Introduction

The treatment of intraarticular distal humerus fractures is subject of continuous debate in the orthopaedic literature [1–13]. They are uncommon, the anatomy is complex, and bone is frequently comminuted [14, 15]. It explains why these fractures pose a significant challenge for the orthopaedic surgeon.

The nowadays debate is related to the type of treatment (open reduction and plate osteosynthesis versus arthroplasty), to the type of plating in case of osteosynthesis (parallel versus perpendicular), and to the surgical approach [3]. Although the posterior approach using the olecranon chevron osteotomy is considered the gold standard [3, 4, 11, 16, 17], the reconstruction of the osteotomy may lead to complications. These complications include delayed union, wound dehiscence, nonunion, malunion, hardware failure, and pain secondary to prominent hardware (Table 1). Alternative approaches to avoid these complications have been reported during the last years, such as the triceps-splitting [9, 18], triceps-reflecting anconeus pedicle [15, 19], the anconeus-flap transolecranon approach [20], and the triceps-sparing approach [21].

To our best knowledge, there is only one recent study evaluating its systematic use for the treatment of distal humerus fractures AO/ASIF type 13-C in the adult patient [21]. In this series of 7 patients, good results were published, and no complication was reported associated to the surgical approach.

Two main questions arise at this point. (1) Does the triceps-sparing approach allow treating these fractures? (2) What are the results and inherent complications related to this approach for the treatment of distal humerus fractures?

In order to answer these two questions, we conducted a prospective study using the triceps-sparing approach to treat AO type 13-C fractures and compared our results with the results obtained in the most recent series treating these types of fractures.

## 2. Materials and Methods

During a period of 3 years (between January 2005 and January 2008), fourteen patients (nine women and five men) with an acute fracture of the distal part of the humerus AO type C underwent an open reduction and internal fixation using the triceps-sparing approach at our institution. The average age of the patients at the time of the surgical procedure was sixty-four years (range from twenty-four to eighty-four).

The patients were informed about the study and gave informed consent, and their data was included in a longitudinal prospective registry. Two patients were lost to followup for reasons unrelated to their elbows. The study group included twelve patients (eight women and four men). The average age of the series was sixty-three years (range from twenty-four to eighty-four).

Fractures were classified on the basis of plain injury radiographs and intraoperative findings according to the Comprehensive Classification of Fractures [14]. According to AO classification, the fractures were classified as 13-C1 in three cases, 13-C2 in two cases and 13-C3 in seven cases. Two of the 13-C3 fractures were open: one type I and one type IIIA fracture according to the Gustilo Open Fracture Classification [27, 28]. One patient presented an ipsilateral diaphyseal ulnar fracture, one patient presented an ipsilateral radial and ulnar diaphyseal fracture, and two patients presented hardware in the olecranon, implanted in a previous surgery. According to the American Society of Anaesthesiologists, 7 patients were classified ASA type II, and 5 patients were classified ASA type III.

Seven out of the twelve fractures were involved in the dominant extremity. A total of six patients had fallen on the floor or ground and two from stairs, and four patients had suffered from a high energy trauma secondary to a motor vehicle accident. Prior to the fracture, all patients could perform daily life activities independently, and no patient reported previous elbow diseases. The time between injury and surgery averaged two days (range from zero to six).

### 2.1. Surgical Technique

The surgical procedures were carried out by the same surgeon (J. A. Fernández-Valencia) through the same technique. All patients were placed in supine position, and the surgery was performed without the use of a tourniquet. The shoulder was placed at 90° flexion and the elbow at 90° flexion. A posterior midline incision with slight radial deviation over the olecranon was used; the ulnar nerve was routinely identified, tagged with a vessel loop, and mobilized proximal and distal to the ulnar tunnel. Triceps tendon was reflected as described by Bryan and Morrey [29].

**Figure 1:**
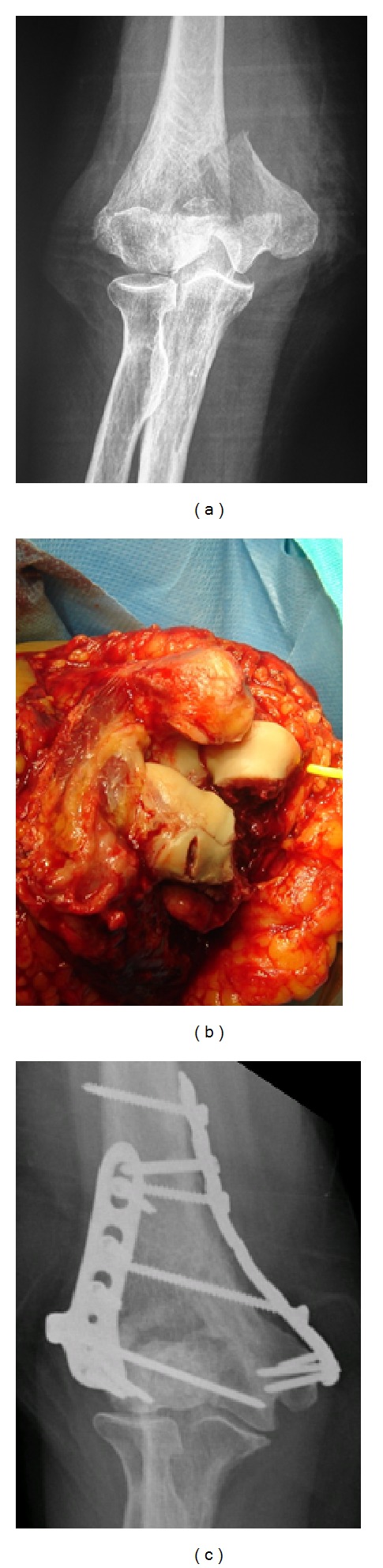
Images of case 10: (a) radiological appearance of the fracture in anteroposterior view; (b) intraoperative image depicting the appearance of the fracture using the Bryan-Morrey triceps-sparing approach; (c) radiological control at the latest follow-up visit, showing union of the fracture, without the need of an olecranon osteotomy.

The fixation was performed reducing the parts of the fracture to either the lateral or the medial column, using the Kirschner wires or screws, to finally complete the reduction of the two constructs under the olecranon (Figure 1). Once reduced, the fracture was stabilized using two 3.5 mm Distal Humerus Plates (DHP, Synthes) orthogonally in all but in two cases. For these two cases, in one elbow (case 1) the fixation was achieved using the Mayo Clinic Congruent Elbow Plates (Acumed), and in the other elbow (case 3), the fracture fixation was achieved using a 3.5 mm Limited Contact-Dynamic Compression Plate ((LC-DCP), Synthes) for the internal column and a 3.5 mm pelvic reconstruction plate for the external column (Synthes). Penetration into the coronoid process fossae was avoided in all cases. The triceps tendon was securely reattached to olecranon with heavy (no. 5), nonabsorbable suture after fracture fixation. This suture was placed through crossed holes in the ulna with a criss-cross stitch in the triceps tendon, and an additional transverse suture was placed to secure the triceps to the tip of the olecranon. At the end of the procedure, the ulnar nerve was transposed subcutaneously for all the cases. No drains were used, and the skin was closed with metallic staples.

### 2.2. Postoperative Management

In nine cases no immobilization was applied, and rehabilitation of the elbows was started immediately as the pain and swelling decreased. Full active assisted movements were allowed, and weight bearing and active movements against resistance were allowed at the 6th week. In two patients the mobilization of the elbows was decided to be delayed for a total of four weeks.

### 2.3. Clinical Evaluation

After a mean followup of 1.7 years (range from twelve to forty-sevenmonths), all the twelve patients underwent a clinical evaluation by an independent observer. Their elbow pain, motion, stability, and function were assessed according to the Mayo Elbow Performance Score [30]. The Mayo Elbow Performance Score ranges from 5 to 100. A score over 90 is considered excellent, between 75 and 89 is good, and between 60 and 74 is fair, and a score under 60 is poor. Strength both in flexion and extension was evaluated comparing to the contralateral side.

Complications after surgery were registered, with special interest in determining the incidence of ulnar nerve damage and the reoperation rate related to complications due to the surgical approach.

### 2.4. Radiographic Analysis

Radiographs of the elbow were taken at the 2nd week and at 2nd, 6th, and 12th months of followup. In all cases the elbow was studied in anteroposterior and lateral views. A final radiograph was taken at the last clinical visit when the followup was larger than 12 months. The radiographs were evaluated to determine union, maintenance of the reduction, implant failure, and heterotopic ossifications (HO). The heterotopic ossifications were classified according to the rating system of Hastings and Graham [31]: class 1 (HO without functional limitation), class 2A (HO with limitation in elbow flexion or extension), 2B (HO with limitation in forearm pronation or supination); 2C (HO with limitation in flexion/extension and pronation/supination), and 3 (ankylosis of the elbow or forearm).

## 3. Results

### 3.1. Clinical Results

Physical examination revealed an average range of motion of 112.8° (range from 85° to 135°). The elbow flexion averaged 125.5° (range from 112° to 135°) and the deficit of elbow extension 14.6° (range from 0° to 30°). There was no limitation of prosupination of the forearm, and all the elbows were stable. The Mayo Elbow Performance Score averaged 93.3 (range from 80 to 100). Nine cases were excellent results, and three were good. Range of motion in AO type C1 had an average of 115° (range from 102° to 130°) classified as good, whereas AO type C3 fractures had an average of 112° (range from 85° to 135°).

Three of the twelve patients presented ulnar nerve paraesthesia. At the latest followup 26 months after surgery only one patient (case 11) recalled to feel a slight and occasional hypoesthesia. The other two patients (cases 5 and 9) recovered spontaneously at the 4th and 6th months, respectively. No failure of the triceps reattachment to the olecranon was found, and all the patients recalled returning to their previous daily life activities without impairment. However, when the flexion and extension strength were compared to the contralateral side, in 2 cases a moderate decrease of strength both in flexion and extension was observed.

One of the patients (case 2) presented with a superficial infection caused by *Pseudomonas aeruginosa*, which was successfully treated with intravenous antibiotics without the need of surgical debridement. No deep infection was documented, and no hardware failure was found. Varus-valgus and posterolateral instabilities were checked at the final examination, and all of the cases were stable. One patient (case 4) underwent a lateral column procedure and removal of the external plate to treat elbow stiffness. The results for each case are summarized in Table 2.

### 3.2. Radiographic Results

In some cases the evaluation of the union was difficult due to the presence of the plates and screws, and inexact views of the distal humerus (not an accurate lateral or anteroposterior view) and the exact time to healing could have been overestimated. However, all fractures showed radiological signs of union at an average of 12 weeks (range from 10 to 14 weeks). Anatomic reconstruction of the articular surface was achieved and maintained in all patients except for one, in which a secondary shift of 2 mm of a trochlear fragment was observed. In one case (case 2) a secondary displacement of a cannulated screw was observed, without clinical implications. In this same case, a heterotopic ossification graded as class 1 was observed.

## 4. Discussion

The experience reported with the use of the triceps-sparing approach to treat distal humerus fracture in adult patients is scant. Some anecdotal reports on the use of the triceps-sparing approach in adults have been performed previously. For instance, in a series of thirty-four complex distal humerus fractures by Sanchez-Sotelo et al. [26], the triceps-sparing approach was performed in two elbows, whereas the TRAP was used in seventeen elbows and the olecranon osteotomy in five. However, the results of those two elbows were not explained in detail. The only series that analyzes exclusively a group of patients treated with this approach has been published recently by Ek et al. [21]. They analyzed the results on 7 patients who managed using this technique. After 2.9-year followup, all fractures had healed and reported a mean score for the MEPS of 83 out of 100, with all 7 patients achieving a good grade. In the present series of 12 patients, after 1.7-year followup, also all the fractures healed, and the mean score for the MEPS has been of 93.3 out of 100. Nine cases were excellent results, and three were good.

In the present study, the triceps-sparing approach allowed to visualize and reduce the fragments properly. It has been demonstrated by Wilkinson and Stanley [32] that the difference of visualization between the triceps-sparing and the olecranon approach is the lack of visualization of an 11% of the surface and that even the olecranon osteotomy leaves a 43% of the surface unseen. In their study they performed an anatomical study on cadaveric elbows comparing the triceps-splitting, triceps-sparing, and olecranon osteotomy approaches with the aim of determining which of them provided the greatest exposure of the distal humeral articular surface. The median exposed articular surface was 35%, 46%, and 57%, respectively. However, this study considered the distal humerus in integrity. In the articular fracture setting, the fragments can be reconstructed to either the lateral or the medial column outside the olecranon, allowing a more extensive visualization during the reduction.

The outcome obtained in the present series of twelve elbows is comparable to that of many other series using the olecranon osteotomy [3, 16] but avoiding the complications related to the osteotomy. On the other hand, the potential complications of the Bryan-Morrey approach must be taken into account, including triceps avulsion or triceps insufficiency, those being complications well described in the setting of total elbow arthroplasty [29, 33]. In fact, in the original series of Morrey and Bryan's initial experience, a 29% incidence of triceps weakness was reported, and 2 patients required reoperation for triceps avulsion [29]. In the present series, two of the twelve patients presented a weakness both in flexion and extension when comparing to the contralateral side, but all were able to perform their previous daily life activities without impairment.

The drawbacks of our study include the limited number of cases and the absence of a control group treated using the olecranon osteotomy. Another limitation of the study is the absence of an objective quantification of muscle strength both for flexion and extension of the elbow. However, the fractures were all articular, were treated by a single surgeon through the same approach, and lastly were evaluated by an independent observer.

As a conclusion, when faced with a complex fracture of the distal humerus, the surgeon could consider avoiding the olecranon osteotomy. Three clinical settings can be favourable to use this approach: those cases in which a total elbow prosthesis might be needed, those cases of ipsilateral diaphyseal fracture, or cases with presence of previous hardware in the olecranon. In our experience, the triceps-sparing approach allowed the correct visualization to perform open reduction and internal fixation, even for C3 fractures, and the outcomes obtained have been satisfactory. Subsequent investigations are mandatory to balance the weight of our results.

## Figures and Tables

**Table 1:** Reported complications related to the olecranon osteotomy approach for the treatment of distal humerus fractures AO type C.

Authors, year	*N*	Nonunion or delayed union	Other complications
Athwal et al. 2009 [2]	17	1 olecranon nonunion	4 wound problems (2 dehiscences and 2 wound breakdowns)
Coles et al. 2006 [4]	70	1 delayed olecranon union	2 early revisions of osteotomy fixation 18 removal of olecranon implants (5 isolated and 13 associated to another procedure)
Doornberg et al. 2007 [22]	19	None	2 wound infections
Elhage et al. 2001 [5]	39	1 olecranon nonunion	1 infection related to the prominent Kirschner wire
Gofton et al. 2003 [6]	17	None	1 plate screw penetrating the proximal radioulnar joint, interfering with forearm rotation, and requiring a second procedure 1 removal of olecranon implant
Greiner et al. 2008 [23]	12	1 delayed olecranon union	None
Gupta and Khanchandani 2002 [7]	13	None	1 wound breakdown and subsequent infection, needing surgical revision 4 proximal migrations of the K-wires needing surgical revision
Holdsworth and Mossad 1990 [24]	57	3 olecranon nonunion	1 septic olecranon bursitis 2 patients needing removal of the Kirchner wires
Kundel et al. 1996 [25]	55	4 olecranon nonunion	None
Liu et al. 2009 [8]	35	None	2 superficial wound infections
McKee et al. 2000 [9]	26	None	3 removal of olecranon implants due to hardware complaints
Pajarinen and Björkenheim 2002 [10]	14	1 olecranon nonunion	None
Ring et al. 2004 [11]	45	None	1 loosening of the wire fixation requiring reoperation (plate fixation) 12 removal of the wires (6 hardware complaints; 1 septic olecranon bursitis; 5 associated to another procedure)
Rübberdt et al. 2008 [12]	11	None	None
Sanchez-Sotelo et al. 2007 [26]	5	None	None
Sané et al. 2009 [13]	14	1 olecranon nonunion	5 bad quality olecranon fixations

*N*: number of distal humerus fractures.

**Table 2:** Clinical features of the series.

Case	Age (years)/sex	Side	Mechanism	Fracture type	Plates	MEPS (months after surgery)	ROM F/E (deg)	Complications
1	71/F	L	MVA	C1	MCCEP	100 (47)	112/10	None
2	84/F	L	Fall	C3*	DHP	95 (14)	130/20	Superficial infection and heterotopic ossification
3	49/F	L	Fall	C1	LC-DCP	85 (12)	128/10	None
4	24/M	L	Fall	C1	DHP	95 (14)	130/20	Elbow stiffness and hardware complaints
5	48/M	R	Fall	C1	DHP	80 (12)	115/30	N. ulnaris paraesthesia
6	73/F	L	Fall	C1	DHP	100 (24)	130/0	None
7	65/M	R	Fall	C3	DHP	100 (12)	120/20	None
8	77/F	R	MVA	C1	DHP	85 (36)	130/15	None
9	55/M	L	MVA	C3**	DHP	100 (18)	135/0	N. ulnaris paraesthesia
10	79/F	R	Fall	C3	DHP	100 (25)	126/0	None
11	49/M	R	MVA	C3	DHP	95 (12)	120/30	N. ulnaris paraesthesia
12	78/F	L	Fall	C3	DHP	85 (12)	130/20	None

M: male; F: female; R: right; L: left; MVA: motor vehicle accident; MCCEP: Mayo Clinic Congruent Elbow Plates (Acumed); LC-DCP: Limited Contact Dynamic Compression Plate (Synthes); DHP: distal humerus plate; MEPS: Mayo Elbow Performance Score; F/E: flexion/extension. *Open fracture type I according to the Gustilo open fracture classification. **Open fracture type IIIA according to the Gustilo open fracture classification.
